# Supplementary material for: Mammalian Orthoreovirus (MRV) Is Widespread in Wild Ungulates of Northern Italy
Source: Viruses. 2021 Feb 3;13(2):238. doi: 10.3390/v13020238 (PMC7913563; doi:10.3390/v13020238)
Supplement: Supplementary file 1 [file viruses-13-00238-s001.pdf]

Supplementary Data

Table S1. Samples selected for viral isolation (n = 11) in the two investigated areas (Sondrio and Parma).

Sample_ID	Host	Sampling area	Gender	Collection year	Age class[1]
**1**	Cervus elaphus	Sondrio	Male	2018-2019	0
**2**	Cervus elaphus	Sondrio	Female	2017-2018	2
**3**	Cervus elaphus	Sondrio	Female	2018-2019	1
**4**	Sus scrofa	Sondrio	Male	2018-2019	1
**5**	Sus scrofa	Sondrio	Male	2019-2020	0
**6**	Sus scrofa	Sondrio	Male	2019-2020	1
**7**	Sus scrofa	Sondrio	Female	2018-2019	2
**8**	Sus scrofa	Sondrio	Female	2019-2020	0
**9**	Sus scrofa	Sondrio	Female	2019-2020	2
**10**	Sus scrofa	Parma	Male	2018-2019	0
**11**	Sus scrofa	Parma	Female	2019-2020	1

[1] Class 0: young; class 1: sub-adults; class 2: adults.
